# Commissioning Tests of Radixact Synchrony Using Patient-Specific 3D-Printed Lung Phantom Inserts

**DOI:** 10.7759/cureus.83532

**Published:** 2025-05-05

**Authors:** Sharon R Gordon, Scott B Crowe, Jemma Walsh, Catherine S Bettington, Tanya Kairn

**Affiliations:** 1 Cancer Care Services, Royal Brisbane and Women's Hospital, Brisbane, AUS; 2 School of Electrical Engineering and Computer Science, University of Queensland, Brisbane, AUS; 3 School of Chemistry and Physics, Queensland University of Technology, Brisbane, AUS; 4 Medical School, University of Queensland, Brisbane, AUS

**Keywords:** ‎3d printing, lung cancer, motion management, radixact synchrony, real-time adaptive radiotherapy

## Abstract

The use of Radixact Synchrony real-time adaptive motion management for lung cancer patients depends on automated detection and delineation of the tumor from surrounding tissues, in addition to the use of surrogate tracking. This functionality should ideally be tested during commissioning using quality assurance phantoms prior to clinical use. However, most dynamic thorax phantoms feature simplistic lung tumors-easily delineated spheres surrounded by low-density tissue. The aim of this project was to design and fabricate more realistic phantoms for Synchrony testing. A selection of eight lung cancer patient CT datasets was retrospectively selected, encompassing variations in size, edge definition, soft tissue attachment, and surrounding fibrosis. Tissues were segmented within 3D Slicer to produce multiple 3D models (in STL format), each corresponding to a range of CT number values. These 3D models were printed at different densities within a larger lung insert that could be inserted into a CIRS Dynamic Thorax Phantom. Mock treatment plans were prepared for these treatments. Replacement lung rods containing more realistic tumors were printed and successfully used for Radixact Synchrony commissioning tests. Results found that across all 3D-printed lung inserts, Radixact Synchrony was able to track motion within 1 mm in both the superior-inferior and left-right directions for a typical lung motion. During erratic breathing motions in the high-frequency breathing trace, Radixact Synchrony was able to approximately pause treatment when the correlation model aged. 3D printing provides an easy and affordable way to extend the functionality of existing phantoms and to assist in the commissioning of novel technologies such as Radixact Synchrony.

## Introduction

Patient motion during treatment can reduce the accuracy of radiotherapy and impact treatment outcomes [1]. Broadly, there are several methods employed to mitigate the impact of respiratory-related motion: motion encompassment, respiratory gating with or without breath holds, motion mitigation by abdominal compression, and real-time motion synchronization techniques, sometimes known as tumor tracking or jaw or multi-leaf collimator tracking [2-6]. The use of motion-encompassing margins, or internal tumor volumes (ITVs), can result in increased dose to surrounding normal tissue [5]. The objective of real-time motion synchronization techniques is to minimize normal tissue exposure while reducing the probability of a geometric miss, with minimal discomfort for patients or a decrease in treatment efficiency [4].

Radixact Synchrony (Accuray, Sunnyvale, USA) is a helical TomoTherapy treatment modality incorporating an on-board kV imaging beam (orthogonal to the treatment beam) and optical surface tracking, which has the capability to perform real-time motion synchronization. For patients treated using the "lung with respiratory tracking" mode of Radixact Synchrony, two to six 2D radiographs are acquired with each rotation of the treatment gantry to monitor the position of the tumor. This data is combined with the position of tracked LED markers placed on the patient's chest wall or abdomen to produce a correlation model able to continually predict the target position, allowing synchronization of field collimation with target motion.

Prior to the clinical release of Radixact Synchrony, it is paramount that comprehensive acceptance and commissioning tests are performed to assess its functionality. Initial commissioning tests within our Radiation Oncology Department at the Royal Brisbane and Women's Hospital (RBWH) involved the characterization of tumor identification and tracking capabilities using a CIRS Dynamic Thorax Phantom (Model 008A, CIRS Inc., Norfolk, VA, USA) with vendor-supplied high-density spherical inserts. Clinically, tumors do not present in perfect contrast with surrounding lung tissue, nor in perfect spherical shapes [7]. Currently, our departmental equipment does not provide the possibility to represent clinical complex structures that move within a dynamic phantom. The aim of this technical report was to design and fabricate lung inserts to replicate clinically complex lung tumor structures as part of commissioning our Radixact Synchrony system.

3D printing refers to a family of manufacturing technologies that fabricate 3D objects by subsequently adding input material in a layer-by-layer method [8]. Polylactic acid (PLA) is one of the most commonly used synthetic polymers for medical applications, owing to its low cost, strength, natural biocompatibility, low toxicity, and nominal density equal to or exceeding water and soft tissue of 1.3 g cm^-3^ [8-10]. Lightweight foaming PLA (PLA-LW) is a 3D printing filament containing a foaming agent that will decompose and release gas when exposed to temperatures within the extrusion nozzle [9]. Decomposition of PLA-LW will create microscopic bubbles (diameter less than 0.1 mm) within the extruded material. By changing the temperature of the extruded material and flow rate to alter the size and quantity of bubbles, the density of the PLA-LW can be modified [9]. Moreover, the density and radiological properties of the PLA and PLA-LW can be modified by varying the infill properties of the extruding material [8-10]. Infill density refers to the ratio of printed material to air within a unit volume [9], where 0% is hollow and 100% is solid extrusion material. The pattern in which the infill material is printed can affect the mechanical and radiological properties [8]. The gyroid infill design has been found to be isotropic when CT imaged [11], while the line and grid design offers the fastest printing time [12]. 3D printing was chosen as a method to design and fabricate complex clinical structures.

## Technical report

Patient selection

**Figure 1:**
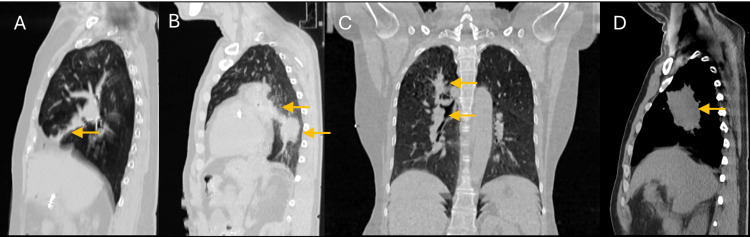
Morphologies of the retrospective selection of patients. Different lung tumor morphologies of retrospectively selected patients: a) tumor with ill-defined edges, b) tumor with mediastinum and soft tissue attachment, c) multiple overlapping lung lesions in a PTV, and d) large tumor with lateral nodule involvement.

Eight patients previously treated within our Radiation Oncology Department at RBWH were retrospectively selected with a range of different morphologies, including ill-defined edges, overlapping lesions, and tumors attached to other structures (Figure 1, Table 1). Clinical contours from their simulation CT were imported into 3D Slicer (version 5.8.1, retrieved from https://www.slicer.org/) [13].

**Table 1:** Lung inserts with their corresponding diagnosis and a morphological description of the gross tumor volume (GTV).

Case number	Diagnosis	Morphology description
1	Non-small cell lung cancer	Very small. Clearly delineated and surrounded by lung tissue.
2	Squamous cell lung cancer	Large. Central location with lateral nodule also included in treatment volume.
3	Localise relapsed classic Hodgkin lymphoma	Attached to the mediastinum.
4	Limited-stage small-cell lung cancer	Very large. Attached to the mediastinum.
5	Colorectal cancer metastasis	Clear edges and central location.
6	Lung adenocarcinoma	Multiple overlapping lesions.
7	Non-small cell lung cancer	Post/medial location with fairly fuzzy edges. Drifts into the mediastinum.
8	Squamous cell lung cancer	Fuzzy edges.

Segmentation 

**Figure 2:**
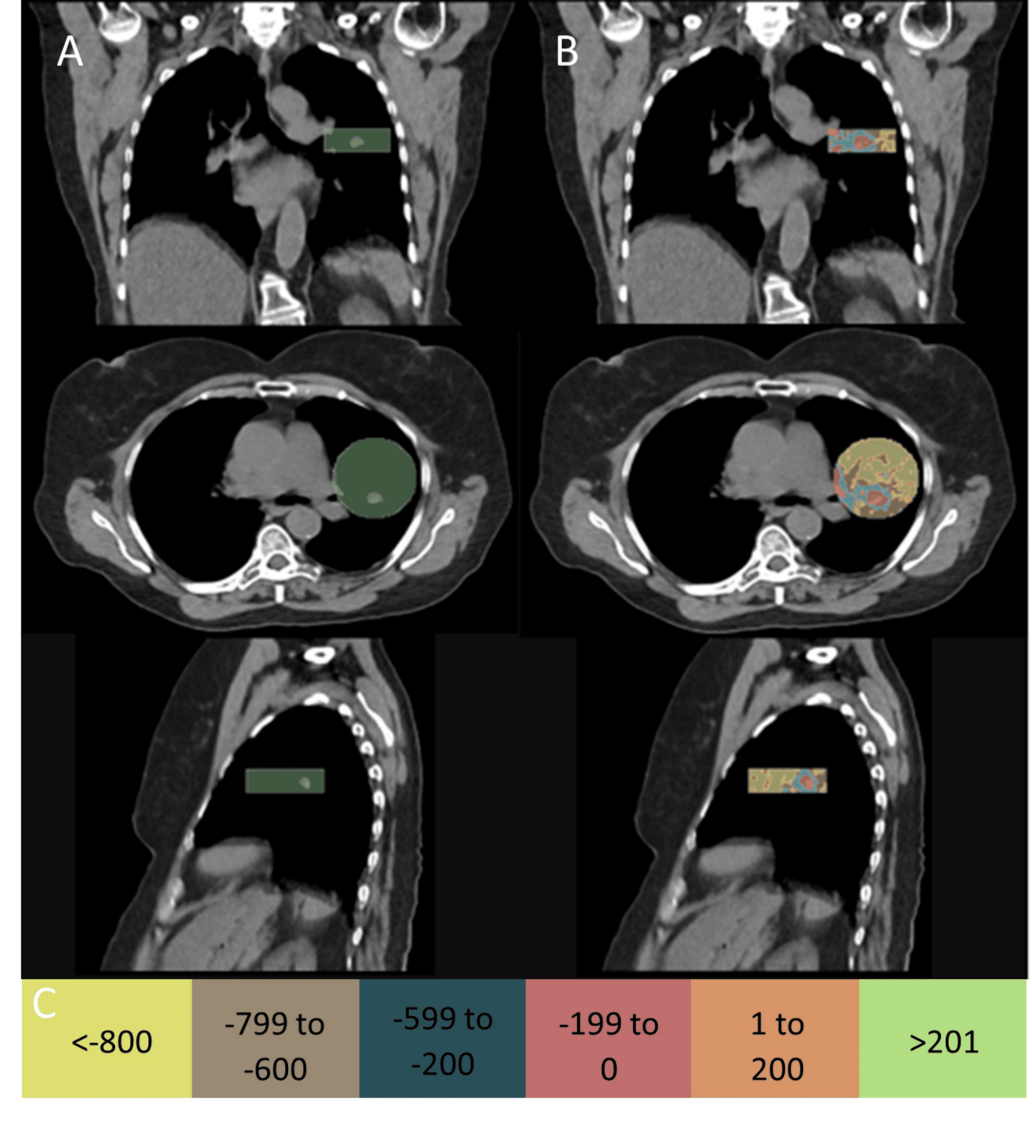
Hounsfield unit windowing and segmentation. a) A 63 mm cylinder radius (same radius as the CIRS dynamic lung insert) drawn around the gross tumor volume. b) CT numbers were windowed into various HU levels: ≤-800, -799 to -600, -599 to -200, -199 to 0, 1 to 200 and ≥200. These HU groups were masked inside the cylinder. c) Color scale for HU windowing.

In 3D Slicer (v5.8.1), a circle with the same diameter as our CIRS Dynamic Thorax lung insert (63 mm) was drawn around the gross tumor volume (GTV) contour and extended an appropriate length to create a cylinder enclosing the GTV and surrounding clinical structures, as shown in Figure 2a. CT numbers (defined in Hounsfield units, HU) were windowed into various groups and masked inside the cylinder structure, as shown in Figure 2b. Common HU windowing thresholds used were ≤-800, -799 to -600, -599 to -400, -399 to 0, 1 to 200, and >201 (Figure 2c). These HU windows were selected following a qualitative assessment of threshold volumes to reduce the presence of volume features with widths less than 2 mm, which were anticipated to be difficult to reproduce at desired densities using 3D printing, due to filament extrusion widths and infill density. Each CT number group with its associated volume was exported as a separate STL file.

Design and fabrication of 3D-printed lung inserts

**Figure 3:**
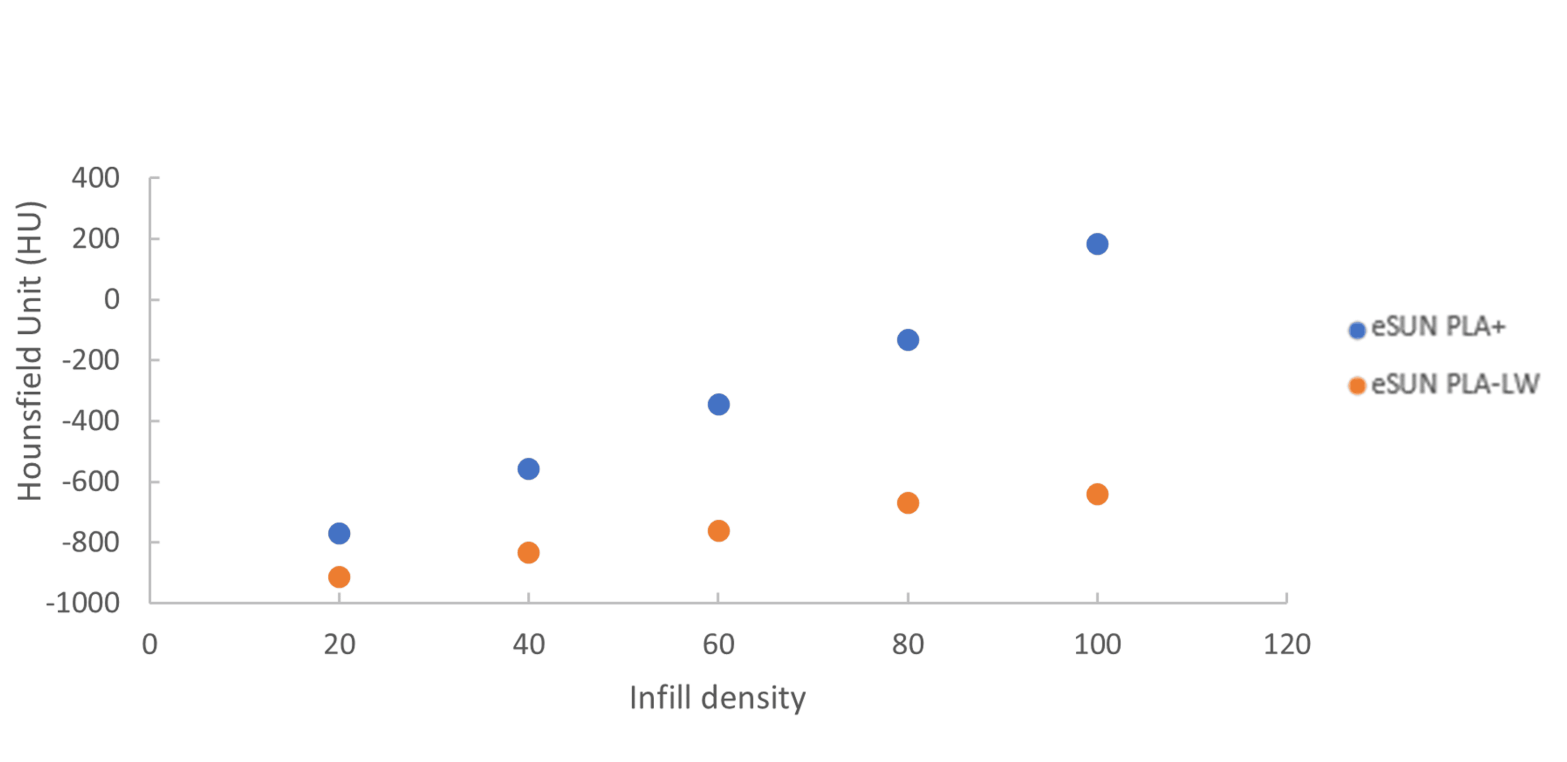
Relationship between the infill density and radiological density. Hounsfield number on our Siemens SOMATOM scanner verses infill density for eSUN PLA-LW and eSUN PLA+ (eSUN Industrial Co., Shenzhen, China) filaments.

The dual-nozzle Raise3D Pro 2 3D printer used in this study has the ability to use two different extrusion materials within a single print. eSUN PLA+ and eSUN PLA-LW (eSUN Industrial Co., Shenzhen, China) were used as the two printing filaments. For all prints, a printing temperature of 220 °C, extrusion width of 0.4 mm, layer height of 0.2 mm, gyroid infill pattern for infill densities less than 90%, grid infill pattern for infill densities greater than 90%, two outer shells, and two single inner shells at interfaces between different filaments were used. To replicate clinical structures when tracked with the Radixact Synchrony system, a relationship between infill density and CT numbers of our materials under kV imaging was established, using an approach described by Crowe et al. [9]. This relationship is shown in Figure 3. It was determined that PLA-LW would be used to print structures with HU less than -600, while PLA+ would be used for structures with HU above -600.

**Figure 4:**
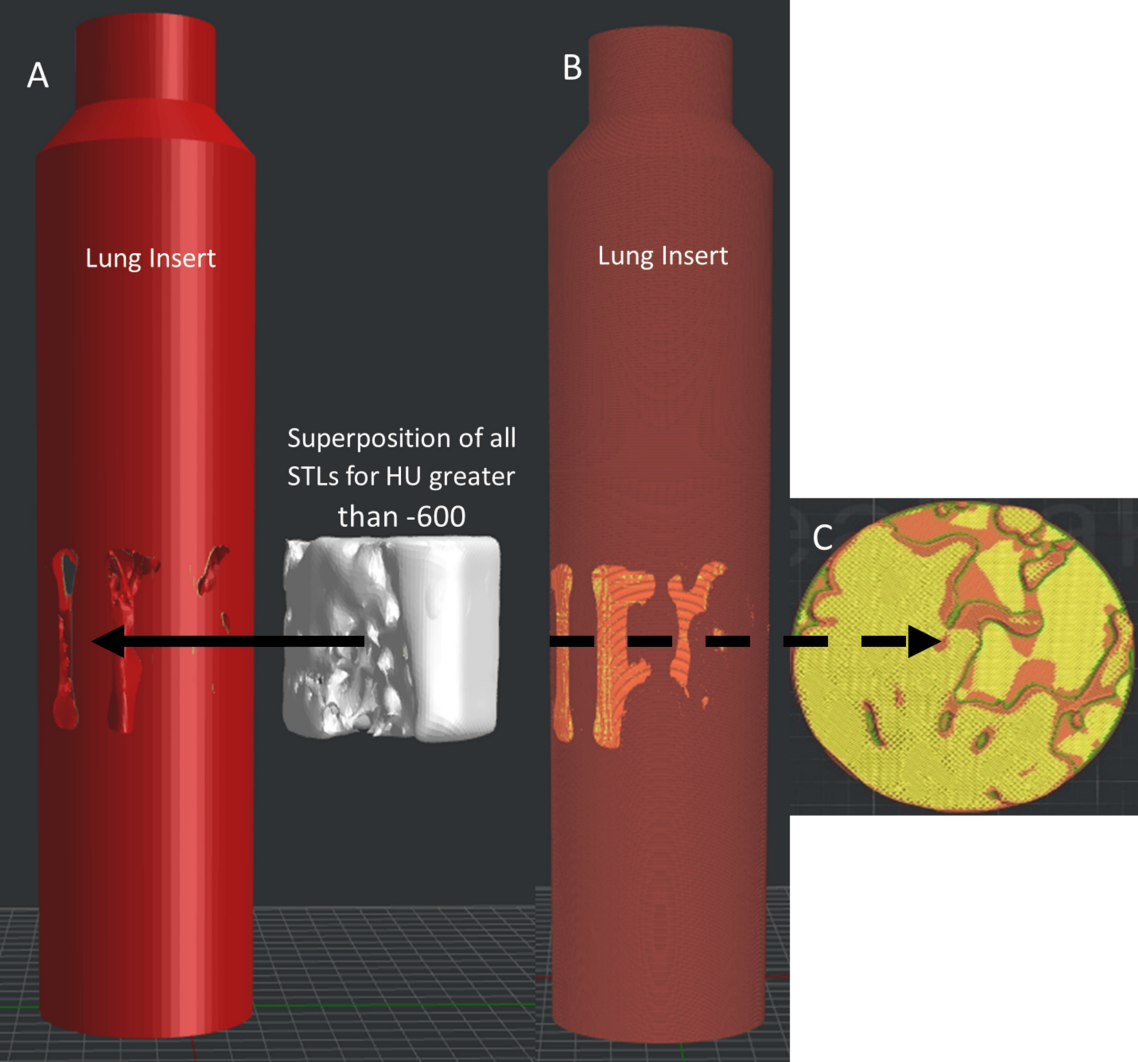
Design of 3D-printed realistic lung inserts for the CIRS Dynamic Thorax Phantom a) Replica of the CIRS lung insert, with a negative of the combined stereolithographs (STLs) for Hounsfield units (HUs) greater than -600. The rod was set to the PLA extruder nozzle, and STLs with HU less than -600 were imported as modifiers. HU groups greater than -200 were imported as modifiers for the PLA-LW nozzle. b) Lung insert ready for 3D printing with corresponding infill densities for both filaments; orange corresponds to PLA-LW and yellow corresponds to PLA+. c) Cross-section of the lung insert with various infill densities and infill patterns for corresponding HU groups.

In ideaMaker (v4.2.3, Raise3D, Irvine, USA), a lung insert with the same dimensions as the CIRS Dynamic Thorax lung insert was replicated. The lung insert was assigned the PLA-LW material and infill density of 50% (Table 2). The STLs for all segmented volumes with HU greater than -800 were imported into ideaMaker and aligned to the center of the insert. Segmented volumes with HU greater than -600 were combined or superposed and used to create both negative space within the PLA-LW lung insert by boolean subtraction and to define the combined volume to be printed with PLA+ and an infill density of 55% (Figure 4). The segmented volumes representing -799 to -600 HU and groups greater than -199 HU were imported as modifiers, or overrides, to the PLA-LW lung insert and combined PLA+ volumes, respectively. The infill densities and patterns used for these overrides are shown in Table 2. The infill density values were determined using Figure 3.

**Table 2:** Summary of the extruder nozzle and infill settings for Hounsfield unit groups used to a print realistic lung insert.

HU window group	Filament material	Infill density	Infill pattern
≤-800	eSUN PLA-LW	50%	Gyroid
-799 to -600	eSUN PLA-LW	75%	Gyroid
-599 to -200	eSUN PLA+	55%	Gyroid
-199 to 0	eSUN PLA+	70%	Gyroid
1 to 200	eSUN PLA+	95%	Grid
≥ 200	eSUN PLA+	100%	Grid

Print times for each 3D-printed lung insert varied from 18 to 34 hours. The cost of PLA-LW is around 30 USD per kg, and the cost of PLA+ is around 15 USD, with the average weight of each filament per 3D print to be 200 g and 30 g, respectively, it can be estimated that the average cost per lung insert was less than 7 USD.

Treatment planning and delivery

**Figure 5:**
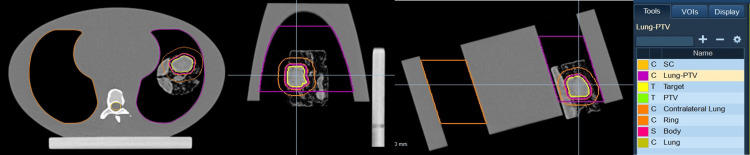
Contouring and treatment planning of a 3D-printed lung insert. Transverse, sagittal, and coronal planes of the treatment plan in the Accuracy Precision treatment planning system. Contours were the lung, contralateral lung, spinal cord (SC), body, gross tumour volume (GTV), and a 2 mm expansion on the GTV to create a planned tumor volume (PTV).

The 3D-printed lung inserts were inserted in the CIRS Dynamic Thorax Phantom and placed onto the CT couch. The phantom was placed at a 16-degree angle between the axis of motion (IEC-Y) and the IEC-X axis to simulate motion in both the IEC-Y (superior to inferior) and IEC-X (right to left) directions. The phantom was CT imaged using a Siemens SOMATOM scanner at 120 kVp (Siemens Healthineers AG, Erlangen, Germany). The CT was imported into the treatment planning system Precision (Accuray, Sunnyvale, US) for contouring and treatment design. A 2 mm margin was expanded from the gross tumor volume (GTV) to create a planned tumor volume (PTV); other contours included the body, lung, contralateral lung, and spinal cord (Figure 5). Notably 2 mm margin is tighter than what is clinically recommended; however, to assess the limitations of Radixact Synchrony, a tighter PTV expansion was used in this study.

**Figure 6:**
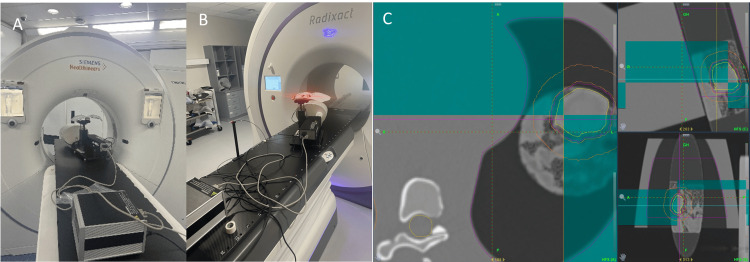
CIRS Dynamic Thorax Phantom setup during the simulation and treatment delivery. a) CIRS Dynamic Thorax Phantoms placed at an angle of 16° from axis of motion and the IEC-X axis to simulate motion both in the IEC-X and IEC-Y directions. b) Exact same phantom setup on the Radixact system. c) Co-registration of simulation and treatment images.

A prescription of 60 Gy in 15 fractions with a field width of 1 cm, a pitch of 0.25, and six imaging angles per gantry rotation was used for all treatment plans. Planned treatment times ranged from 154.5 seconds to 559.5 seconds, and gantry periods ranged from 14 seconds to 20 seconds. The specific angle of each image was qualitatively assessed using the Score Wheel, with all imaging angles across all inserts, except for one image angle in insert 3, obtaining an optimal green score. The CIRS phantom was placed on the TomoTherapy couch at an angle of 16° from the axis of motion and aligned to in-room lasers, to simulate motion of the lung inserts in both the IEC-X and IEC-Y planes (Figure 6a). Three LEDs were placed on the dynamic chest wall, and one was placed on the support arm as shown in Figure 6b. The setup was imaged and co-registered to the treatment plan (Figure 6c). Two breathing motions, a typical and a high-frequency trace, were imported into the CIRS Dynamic Thorax Phantom to replicate the breathing motion of the phantom and chest wall surrogate [14]. Radixact Synchrony was set up to build a correlation model, and once a model was built, treatment commenced. Each lung insert underwent one fraction of typical and one fraction of high-frequency breathing motion. Any treatment pauses due to model aging were noted.

Analysis of tracked motion 

After treatment was completed, the motion data were exported and compared against the typical and high-frequency motion traces in the Accuray MDX tool. 

Tracking performance

**Figure 7:**
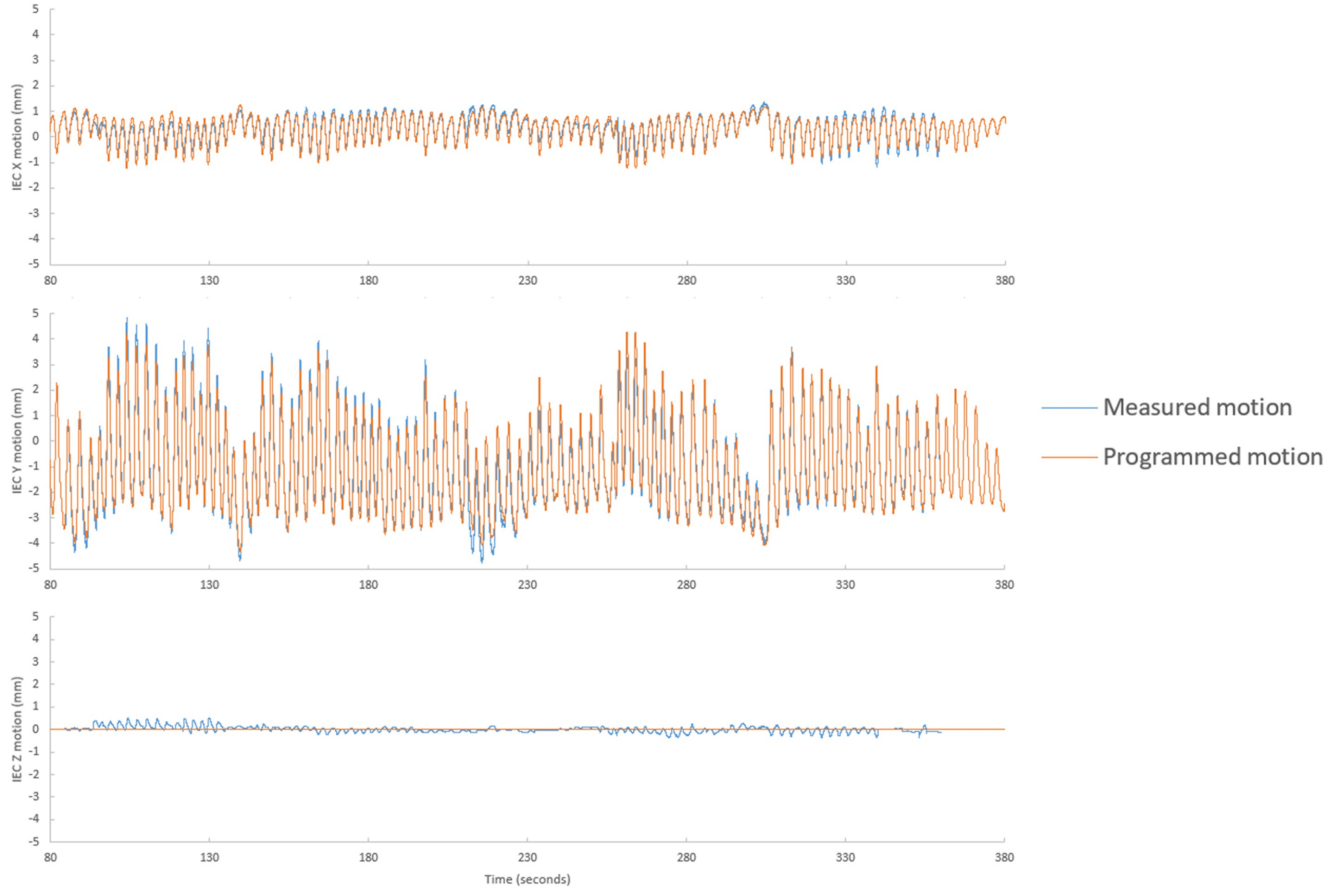
Tracked verses phantom motion for typical lung motion. Motion detected by Radixact Synchrony system (blue) against the programmed motion of a typical breathing motion on the CIRS Dynamic Thorax Phantom (orange).

Figure 7 shows the comparison in IEC-X, IEC-Y, and IEC-Z directions for typical lung motion. Notably, although no IEC-Z motion was simulated with our current phantom setup, it was analyzed to assess if there was any incorrect extrapolation from motion in IEC-X and IEC-Z planes. It was found that across all the lung inserts, typical lung motion had maximum disagreement of less than 1 mm in both the IEC-X and IEC-Y directions. Figure 7 highlights that the median absolute disagreement was less than 1 mm throughout the entire fraction. Only insert 2 experienced a treatment pause due to model ageing.

**Figure 8:**
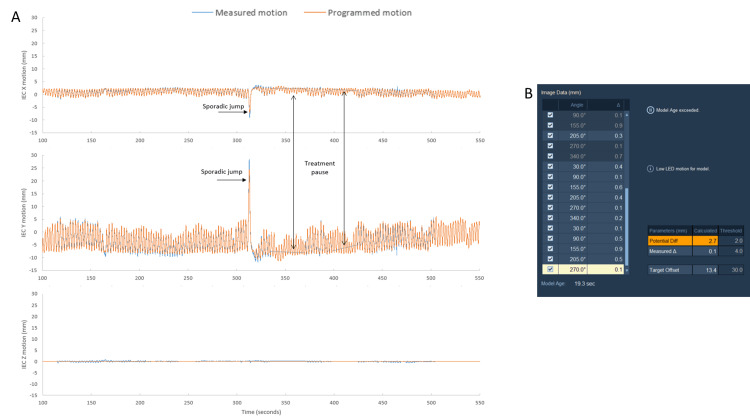
Tracked verses phantom motion for high-frequency lung motion A) Motion detected by the Radixact Synchrony system (blue) against the programmed motion of a high-frequency breathing motion on the CIRS Dynamic Thorax Phantom (orange) in the IEC-X, IEC-Y, and IEC-Z directions. Radixact Synchrony paused treatment after the model aged. Pausing was observed shortly after a sporadic jump in the breathing motion. B) Screenshot from the Radixact Synchrony treatment console during the first treatment pause, showing aging of the lung correlation model.

**Figure 9:**
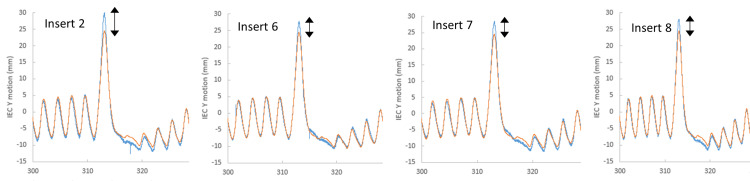
Tracked motion of high-frequency sporadic jump across various lung inserts. The sporadic jump in the high-frequency lung trace, tracked across the different lung inserts with varying morphology. Similar levels of disagreement in the superior-inferior directions.

High-frequency lung motion exhibited greater levels of maximum disagreement in both the IEC-X and IEC-Y directions (Figure 8). There is a sporadic jump in lung motion during the high-frequency trace, wherein it was observed that Radixact Synchrony tracked this erratic motion across the different lung inserts (Figure 9). Figure 8 illustrates that the median disagreement throughout the fraction, apart from the sporadic jump in motion, is less than 1 mm. Table 3 summarizes the maximum disagreement portraying the worst-case scenario across all lung inserts during typical and high-frequency motion.

**Table 3:** Disagreement in the IEC-X and ICE-Y directions during typical and high-frequency lung motions. Any treatment pauses due to model ageing were also noted.

Case number	Maximum disagreement between measured and programmed motion with typical lung motion			Maximum disagreement between measured and programmed motion with high frequency lung motion		
	Treatment pause	IEC-X	IEC-X	Treatment pause	IEC-X	IEC-Y
1	No	<1 mm	<1 mm	No	2 mm	3mm
2	Yes	<1 mm	<1 mm	Yes	2 mm	4mm
3	No	<1 mm	<1 mm	No	2 mm	2 mm
4	No	<1 mm	<1 mm	No	2 mm	3 mm
5	No	<1 mm	<1 mm	No	1 mm	3 mm
6	No	<1 mm	<1 mm	Yes	1 mm	2 mm
7	No	<1 mm	<1 mm	Yes	2 mm	3 mm
8	No	<1 mm	<1 mm	Yes	2 mm	4 mm

## Discussion

3D printing to fabricate clinically complex structures

3D printing has been demonstrated to be a successful method for fabricating clinically complex structures, with scans from our Siemens SOMATOM Confidence CT scanner and kV imaging on our Radixact Synchrony system showing the lung inserts to be similar to their respective clinical contours. Madamesila et al. [15] reported a similar capability of 3D printing to fabricate more complex human structures in a phantom. They were able to replicate low-density lung structures to evaluate heterogeneity modelling of the dose calculation algorithms used in the Eclipse treatment planning system [15]. Importantly, 3D printing was able to extend the functionality of our existing CIRS Dynamic Thorax Phantom to test the respiratory system without fiducial mode with more clinically representative structures. Programmed motion phantoms have been described in previous studies for performing commissioning and quality assurance on motion management systems. Dunn et al. [16] described a method using a programmable motion phantom that enables test patterns and patient motion to be replicated in two dimensions with a decoupling of the chest-wall and insert motion, similar to the method described in this technical report.

Radixact Synchrony tracking

Radixact Synchrony was able to build a correlation model when lung inserts moved in typical or high-frequency lung motion. Variation in the amount of imaging and time taken to build a model was observed across the different lung inserts. Potential difference and measured difference thresholds were set to 2 and 4, respectively; however, if a treatment was paused and a model could not be rebuilt, these thresholds could be increased to facilitate Radixact Synchrony to rebuild a correlation model and resume treatment. Thresholds for potential and measurement differences will therefore need to be determined prior to clinical use.

For all typical lung motions, the agreement between tracked motion and phantom motion was within 1 mm in both the IEC-X and IEC-Y directions. Similarly, Ferris et al. [17] analyzed the statistics of amplitude and period tracking errors between the Synchrony‐predicted motion and the phantom motion. Results showed δ_^RMS^_ of <1.0 mm and δ_95%_ of <2.5 mm for all lung cases. This level of agreement in tracked motion highlights the benefits of real-time motion synchronization and the potential to reduce margins on ITVs or PTVs. Similarly, using a cylindrical target, Schnarr et al. [18] also highlighted the benefits of real-time motion synchronization when comparing the dose profiles between a plan with motion management against a plan without motion management. Results found widening of the dose profiles and a decreased peak dose difference of 30% when no motion management was applied [18]. This is in contrast to the peak dose difference of 2% when motion management was applied [18]. With the trend moving toward hypofractionation regimes, motion management and reducing healthy tissue exposure will become increasingly important [19]. Our data suggests that Radixact Synchrony can greatly benefit patients who can perform reproducible breathing motions across the entirety of their treatment.

The high-frequency breathing motion showed higher levels of maximum disagreement between the tracked versus phantom motion and triggered treatment pauses across different lung inserts. Importantly, a treatment pause was observed straight after a sporadic jump in motion that caused the correlation model to age. Looking across all lung inserts with various morphologies and target volumes for the correlation model, the sporadic jump was tracked with similar levels of disagreement in both the IEC-X and IEC-Y directions (Figure 8). Similarly, Tse et al. [20] performed dosimetric validation using programmed lung traces that exhibited sporadic jumps (maximum amplitude of 10 mm) using a stereotactic phantom with a gamma analysis criterion of 3%/2 mm and a 10% low dose threshold. Results showed 99.49% for a phantom on a static motion platform with no Synchrony tracking, 97.31% for a phantom on a moving platform with Synchrony lung tracking with respiratory modelling, and 38.18% for a phantom on a moving platform with no Synchrony tracking [20]. A potential for maximum disagreement during the sporadic jump in our study could be attributed to insufficient image sampling, wherein the time between two consecutive 2D radiographs was not able to capture the magnitude of motion accurately. The present study did not optimize the imaging; however ,clinically, the optimal imaging angles and frequency can be manipulated by planning staff or at the point of treatment delivery by treatment staff to ensure maximum visibility of tumor volumes. Imaging angles and frequency of intrafraction verification images can be individually optimized to account for distinct tumor presentations to further improve the performance of Radixact Synchrony tracking and minimize the impact of small variations in breathing pattern over long treatment delivery times.

## Conclusions

3D printing provided an easy and affordable way to extend the functionality of our existing CIRS Dynamic Thorax Phantom to replicable clinical complex structures as part of commissioning Radixact Synchrony. We were able to assess the capabilities of the Radixact Synchrony correlation model across a wide range of morphologies. Radixact Synchrony was able to track motion within 1 mm in both the superior-inferior and left-right directions for a typical lung motion. During high-frequency and erratic breathing motions, Radixact Synchrony was able to approximately pause treatment when the correlation model aged. Patients who exhibit sporadic changes in their breathing cycles can still benefit greatly from Radixact Synchrony, where rebuilding a model and commencing treatment has been shown to be a seamless process. 
